# Links between Disease Severity, Bacterial Infections and Oxidative Stress in Cystic Fibrosis

**DOI:** 10.3390/antiox11050887

**Published:** 2022-04-29

**Authors:** Sabina Galiniak, Mateusz Mołoń, Marta Rachel

**Affiliations:** 1 Institute of Medical Sciences, Medical College of Rzeszów University, Rzeszów University, Warzywna 1a, 35-310 Rzeszów, Poland; 2 Department of Biology, Institute of Biology and Biotechnology, Rzeszów University, Zelwerowicza 4, 35-601 Rzeszów, Poland; mmolon@ur.edu.pl; 3 Department of Allergology and Cystic Fibrosis, State Hospital 2 in Rzeszów, Lwowska 60, 35-301 Rzeszów, Poland

**Keywords:** bacterial infection, disease severity, protein oxidation, lipid peroxidation, total antioxidant capacity

## Abstract

Cystic fibrosis (CF) is one of the most common, yet fatal genetic diseases in Caucasians. The presence of a defective CF transmembrane conductance regulator and the massive neutrophils influx into the airways contribute to an imbalance in epithelial cell processes and extracellular fluids and lead to excessive production of reactive oxygen species and intensification of oxidative stress. The study included 16 controls and 42 participants with CF aged 10 to 38. The products of protein oxidation, total antioxidant capacity (TAC) and markers of lipid peroxidation were estimated in the serum of the subjects. Furthermore, we compared the level of oxidative stress in patients with CF according to the severity of disease and type of bacterial infection. Thiol groups and serum TAC decreased significantly in patients with CF (*p* < 0.05). Elevated levels of 3-nitrotyrosine, malondialdehyde and 8-isoprostane were observed in CF subjects (*p* < 0.05). Furthermore, as the severity of the disease increased, there was a decrease in the thiol groups and TAC levels, as well as an increase in the concentration of 3-nitrotyrosine and 8-isoprostane. CF participants infected with *Pseudomonas aeruginosa* had elevated 3-nitrotyrosine concentration levels (*p* < 0.05), while those infected with *Staphylococcus aureus* noted a decrease in thiol groups (*p* < 0.05). Elevated levels of oxidative stress markers were found in the serum of CF patients. Furthermore, oxidative stress progressively increased over the years and along with the severity of the disease. The presence of bacterial infection with *P. aeruginosa* or *S. aureus* had a slight effect on oxidative stress, while co-infection by two species did not affect the level of oxidative stress.

## 1. Introduction

Cystic fibrosis (CF) is a multisystem disease caused by certain mutations causing defective or absent CF transmembrane conductance regulator (CFTR) protein, the main function of which is the transepithelial anions transport. Hence, CF is characterized by impaired secretion of the exocrine glands and affects many organs, especially the digestive and respiratory systems. The glands produce thick mucus, which provides an ideal environment for the continuous development of respiratory infections, especially *Pseudomonas aeruginosa* and *Staphylococcus aureus*, which are associated with poor clinical outcomes [1,2]. CFTR deficiency causes oxidative stress in the airway epithelium of CF, affecting multiple bioactive lipid metabolic pathways, which are likely to play a role in lung disease progression [3]. Moreover, many comorbidities emerging with the increasing patient survival times and improved quality of care lead to exacerbated production of reactive oxygen species (ROS), exceeding the levels required for optimal physiological functioning [4,5,6]. Overgeneration of ROS results in a structural modification of cellular proteins, leading to changes in their function and aggregation, and therefore, cell dysfunction and cellular processes impairment [7]. Accordingly, the implication of oxidative stress in the complex pathophysiology of CF is widely accepted [8,9]. Increased markers of oxidative stress were observed in the serum, plasma, exhaled breath condensate and sputum of CF patients [10,11,12,13]. The purpose of our study was to compare oxidative stress markers in the serum of patients with CF classified according to the severity of the disease as mild, moderate or severe CF. Secondly, we compared the level of oxidative stress in relation to the infection. For this purpose, we divided patients into four groups depending on the bacterial infection: those infected with *P. aeruginosa*, those infected with *S. aureus*, those co-infected by the two species and those uninfected. Finally, we tried to assess the correlations between markers of oxidative stress and the demographic and clinical parameters of the patients. To our knowledge, this is also the first report presenting the level of 4-hydroxy-2-nonenal and the ability of human serum albumin to bind cobalt in CF patients.

## 2. Materials and Methods

### 2.1. Ethical Issues

The study protocol was approved by the Bioethics Committee of the University of Rzeszow (Poland) (12/01/2021). All procedures performed in studies involving human participants were in accordance with the ethical standards of the institutional and/or national research committee and with the Declaration of Helsinki of 1964 and its subsequent amendments or comparable ethical standards. Written informed consent was obtained from patients and legal guardians, in the case of pediatric patients.

### 2.2. Study Group

A single-center study was conducted in a sample of 42 CF patients between the ages of 10 and 38, as well as in control subjects (*n* = 16). Participants were recruited from the Department of Allergology and Cystic Fibrosis, Provincial Hospital No. 2, Rzeszow, Poland, from February 2021 to October 2021. The study involved patients with CF with a confirmed diagnosis based on the determination of sweat chloride, genetics and immunoreactive trypsin test in neonatal age (patients born in or after 2009). The next criteria for enrolling patients in the study were the following: forced expiratory volume in the first second (FEV_1_) greater than 35% of the predicted stable pulmonary disease, as defined by both clinical impressions and no hospitalizations in the 30 days prior to screening. Exclusion criteria were also as follows: heart failure, psychiatric disorder, post-solid organ transplantation, oral corticosteroids treatment, liver disease, infection with *Burkholderia cepacia* or *Stenotrophomona maltophilia* and *Aspergillus*. Additionally, patients were excluded if they were unable to perform spirometry and refused to participate in the study. Due to these reasons, 5 patients were excluded from the study. All CF patients suffered from pancreatic insufficiency and received pancreatic enzyme replacement therapy. (Creon 25000, Solvay Pharmaceutical Inc., Marietta, GA, USA). Patients were also treated with human DNase I recombinant (Pulmozyme, Genentech Inc., San Francisco, CA, USA; one 2.5 mg ampoule inhaled once daily using a nebulizer), fat-soluble vitamins in the form of ADEK tablets (Scandipharm, Birmingham, AL, USA), supplemental nutrition drinks (Nutrison Protein Plus, Nutricia, Poland) and inhalation of 3–10% sodium chloride 3–4 times daily. CF subjects were divided into three groups based on the severity of their disease based on the results of FEV_1_—mild disease (FEV_1_ > 75% predicted, *n* = 27), moderate disease (FEV_1_ from 45% to 75% predicted, *n* = 9) and severe disease (FEV_1_ < 45% predicted, *n* = 6). Additionally, patients were also divided into 4 groups according to bacterial infection: (1) *P. aeruginosa* (*n* = 10), (2) *S. aureus* (*n* = 13), (3) co-infection with *P. aeruginosa* and *S. aureus* (*n* = 9), and (4) no infection (*n* = 10). Healthy subjects aged 10–38 years were recruited at the same time from the local clinic. The control group consisted of sex-matched volunteers with no diseases in the medical history or physical examination (inclusion criteria: no family history of CF, allergy, and other chronic disease, no clinically significant abnormalities in blood chemical assessments, and hematologic assessments that included complete blood count; no use of any antioxidant vitamins). The volunteers had not taken any medications 30 days before the study. All participants in the control group had normal pulmonary function tests. All participants had anthropometric measurements. BMI was calculated as kg/m^2^.

### 2.3. Materials

All basic reagents were purchased from Sigma-Aldrich (Poznan, Poland), unless indicated otherwise. The 3-nitrotyrosine enzyme-linked immunosorbent assay (ELISA) kit was supplied by Immunodiagnostik AG (K7829, Bensheim, Germany). The 8-isoprostane ELISA kit was supplied by Cayman Chemical (516351, Ann Arbor, MI, USA). The 4-hydroxy-2-nonenal (4-HNE) ELISA kit was supplied by Wuhan Fine Biotech Co., Ltd. (EU0187, Wuhan, China). Absorptiometric measurements were performed on a Tecan Infinite 200 PRO multimode reader (Tecan Group Ltd.; Männedorf, Switzerland). All reagents used were of analytical reagent grade. Measurements were taken in triplicate, unless indicated otherwise.

### 2.4. Spirometry

All CF and control subjects performed spirometry using a standard spirometry device (Lungtest 1000, MES, Kraków, Poland) according to recommendations [14]. We calculated the mean value of the last half year for forced vital capacity (FVC), FEV_1_, FEV_1_/FVC expressed as a percentage of the predicted value for age and sex.

### 2.5. Sputum Collection

The agent used for sputum induction and collection was hypertonic saline, and deep throat swab specimens were cultured for bacterial pathogens. Bacterial and fungal infections were classified according to the criteria for *P. aeruginosa* infections, and those criteria were systematically applied to all bacterial/fungal species included in this study [15]. Antimicrobial drug susceptibility tests were performed by agar dilution according to the Institute of methodology of the Clinical and Laboratory Standards Institute [16]. Chronic colonization by *P. aeruginosa* was defined by at least 3 positive sputum tests for this bacterium or the continuous presence of this microorganism in the sputum over 1 year prior to the study. Today, *B. cepacia*, *P. aeruginosa* and *S. aureus* are the most important infectious agents in CF patients. At our center, there are only two patients chronically infected with *B. cepacia,* and therefore, they were excluded from the study.

### 2.6. Blood Sampling

Blood samples were obtained between 8 am and 10 am after fasting overnight and were put into blood collection tubes. Next, the samples were centrifuged (1500× *g*, 10 min, 4 °C), and the serum obtained was aliquoted and frozen at −80 °C until further analysis. The serum samples were not stored for more than 3 months and were thawed at room temperature only once during the analysis.

### 2.7. Blood Counts and Serum Analysis

Blood morphology was performed by using a hematology analyzer (Siemens Healthineers, Erlangen, Germany). Concentration of C-reactive protein (CRP) was estimated using the dry chemistry immunological method on a VITROS 250 analyzer (Ortho Clinical Diagnostics, Johnson and Johnson, Raritan, NJ, USA). Direct potentiometric measurement of Na^+^, K^+^ and Cl^−^ in serum with liquid ion-exchange electrode was performed.

### 2.8. Biochemical Procedures

#### 2.8.1. Protein

The protein concentration was estimated using the method of Lowry et al. [17].

#### 2.8.2. Advanced Oxidation Protein Products

Advanced oxidation protein products (AOPP) were estimated by the method of Witko-Sarsat et al. [18]. AOPP concentration is expressed in nmol chloramine-T equivalents/mg protein.

#### 2.8.3. Thiol Group

The content of thiol groups was estimated by the method of Ellman [19]. The thiol group content was calculated on the basis of a standard curve using glutathione as a standard and expressed in mmol/L.

#### 2.8.4. Amadori Product

The content of the Amadori product was estimated using the method of Johnson et al. [20]. The Amadori products were calculated using an extinction coefficient of 12,640 M^−1^ cm^−1^ for monoformazan [21]. Measurements were taken in duplicate.

#### 2.8.5. 3-Nitrotyrosine

The 3-nitrotyrosine concentration was evaluated with the 3-nitrotyrosine ELISA kit (Immundiagnostik AG), according to the manufacturer’s instruction.

#### 2.8.6. Total Antioxidant Capacity with ABTS

Total antioxidant capacity of serum with ABTS^•^ was estimated by using the method of Re et al. [22]. The results were expressed in Trolox equivalents (μmol TE/L).

#### 2.8.7. Total Antioxidant Capacity with FRAP

Ferric reducing antioxidant power assay (FRAP) was determined colorimetrically by measuring the ferric reducing capacity of serum samples. Ethanol solutions with known concentrations of Trolox were used for calibration. The results were expressed in Trolox equivalents (μmol TE/L) [23].

#### 2.8.8. Malondialdehyde

The concentration of malondialdehyde (MDA) was estimated using the method of Yagi [24] and expressed as μmol/L. The results were calculated using an absorption coefficient for MDA of 1.56 × 10^5^ M^−1^ cm^−1^.

#### 2.8.9. 8-Isoprostane

The 8-isoprostane concentration was evaluated with the 8-isoprostane ELISA kit (Cayman Chemicals, Ann Arbor, MI, USA), according to the manufacturer’s instruction.

#### 2.8.10. 4-Hydroxy-2-nonenal

The 4-HNE concentration was evaluated with the 4-HNE ELISA kit (Wuhan Fine Biotech Co., Ltd., Wuhan, Hubei, China), according to the manufacturer’s instruction.

#### 2.8.11. Albumin Cobalt Binding

Albumin cobalt binding levels were measured by spectrophotometry using Bar-Or’s method [25] and reported in absorbance units (ABSU). Measurements were taken in duplicate.

## 3. Results

A total of 20 female and 22 male participants with CF were enrolled in this study. At the same time, 10 healthy females and 6 males were recruited into the control group. The basic characteristics, clinical laboratory values and lung function indices for CF patients and healthy participants are shown in Table 1.

Homozygosity for ΔF508 was confirmed in 32 patients, while heterozygosity for ΔF508 was found in 10 patients. There were no differences in the age and height (*p* > 0.05). However, statistically significantly lower weight (*p* < 0.05) and BMI (*p* < 0.01) occurred in patients with CF compared to healthy subjects. The clinical hematological results were similar between the CF group and healthy subjects, except for the elevated levels of white blood cells and the percentage of basophils. The concentration of C-reactive protein (CRP) also increased in CF group when compared to healthy controls. Lung function measured by spirometry was worse in CF patients than in healthy participants. Analysis of the results of spirometry allowed for the division of patients with CF according to the severity of the disease into mild (64.3%), moderate (21.4%) and severe (14.3%). Additionally, the CF participants were divided into four groups according to bacterial infection. The first group consisted of patients infected with *P. aeruginosa* (23.8%), the second was infected with *S. aureus* (31%), and the third group was infected with both bacteria (21.4%). Additionally, we included subjects with CF who were uninfected (23.8%).

Table 2 shows the results of the determination of oxidative stress markers in the whole group of patients with CF compared to the control group.

**Figure 1:**
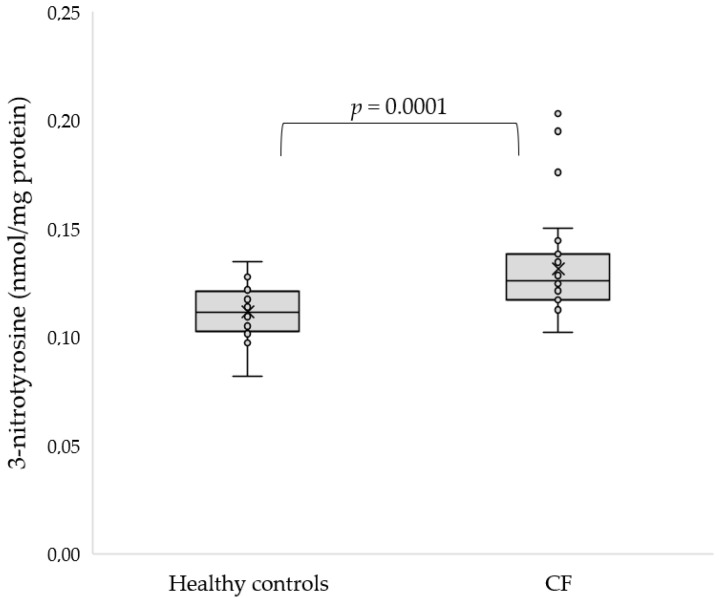
Concentration of 3-nitrotyrosine in serum of CF patients as compared to the control group.

One of the most frequently determined markers of protein oxidative modification is advanced oxidation protein products (AOPP). However, there were no differences in AOPP concentration between the groups (*p* = 0.479). Thiol groups in proteins are vulnerable to oxidative damage. Our study indicates that the concentration of thiol groups was significantly lower in CF participants compared to control subjects (491.57 ± 86.34 vs. 591.65 ± 67.8 µmol/L, *p* = 0.0003). Furthermore, reducing sugar can react non-enzymatically with protein amino groups to form Amadori products that generate ROS in the presence of transition metals and molecular oxygen. There were no differences in concentration in Amadori products between CF and control subjects (*p* = 0.741). In addition, 3-nitrotyrosin is one of the promising biomarkers of oxidative stress, which is formed by the nitration of protein and free tyrosine residues by reactive peroxynitrite molecules. The 3-nitrotyrosine concentration was significantly elevated in CF patients compared to healthy subjects (0.13 ± 0.02 vs. 0.11 ± 0.01 nmol/mg protein, *p* = 0.0001, Figure 1).

**Figure 2:**
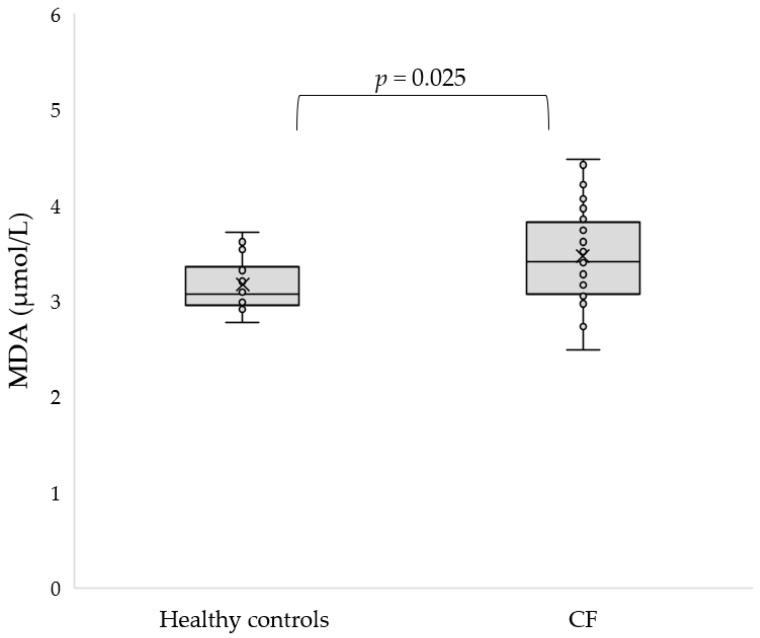
Concentration of MDA in the serum of CF patients as compared to the control group.

In addition, we used two methods to estimate the total antioxidant capacity (TAC) of the serum of the participants. The TAC of the blood serum measured by ABTS^•^ did not show statistically significant differences with respect to the healthy controls (*p* = 0.16). However, the TAC measured by FRAP was significantly lower in CF patients compared to controls (151.6 ± 28.92 vs. 170.68 ± 16.72 µmol TE/L, *p* = 0.023). Lipids are very sensitive to ROS attack, and to date, malondialdehyde (MDA), 8-isoprostane and 4-hydroxy-2-nonenal (4-HNE) are the main biomarkers for lipid peroxidation assessment. An increased concentration of MDA was found in patients with CF (3.47 ± 0.5 vs. 3.17 ± 0.28 μmol/L, *p* = 0.025, Figure 2).

**Figure 3:**
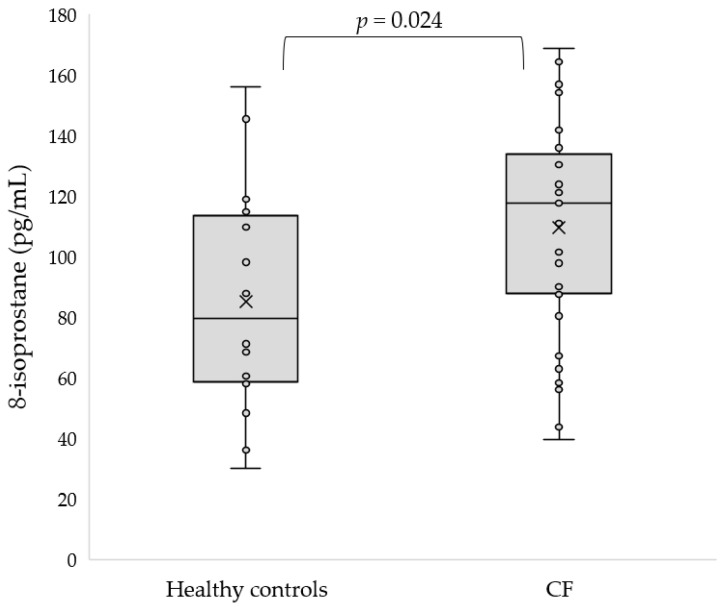
Concentration of 8-isoprostane in serum of CF patients as compared to the control group.

Similarly, the 8-isoprostane concentration was elevated in CF subjects with respect to the healthy controls (110.71 ± 35.74 vs. 85.18 ± 37.33 pg/mL, *p* = 0.024, Figure 3).

However, the concentration of 4-HNE was increased in CF but not significantly (*p* = 0.501). Finally, we employed the estimation of albumin cobalt binding to assess cobalt binding affinity for albumin as a result of modification of the specific cobalt binding site by ROS. We found elevated albumin cobalt binding in CF patients compared to controls (*p* = 0.038).

Table 3 shows the differences in markers of oxidative stress in CF patients depending on the severity of the disease.

**Figure 4:**
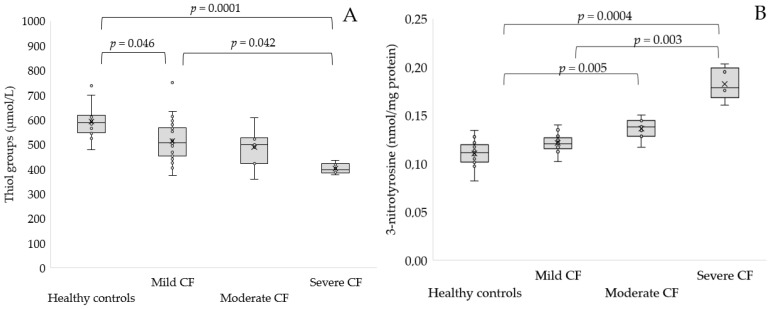
Concentration of thiol groups (**A**) and 3-nitrotyrosine (**B**) in serum of patients with mild, moderate and severe CF as compared to the control group.

No statistically significant differences were found with respect to controls in the concentration of AOPP, Amadori products, MDA, 4-HNE and albumin cobalt binding in any group of patients. However, a lower concentration of thiol groups was found in severe CF compared to controls (*p* = 0.0001) and mild CF (0.4 ± 0.02 vs. 0.52 ± 0.09 mmol/L, *p* = 0.042, Figure 4A). Furthermore, a reduced concentration of thiol groups was observed in mild CF with respect to healthy subjects (*p* = 0.046). A significantly elevated concentration of 3-nitrotyrosine was found in participants with severe CF compared to mild CF (0.18 ± 0.02 vs. 0.13 ± 0.03 nmol/mg protein, *p* = 0.005, Figure 4B) and healthy controls (*p* = 0.0004).

**Figure 5:**
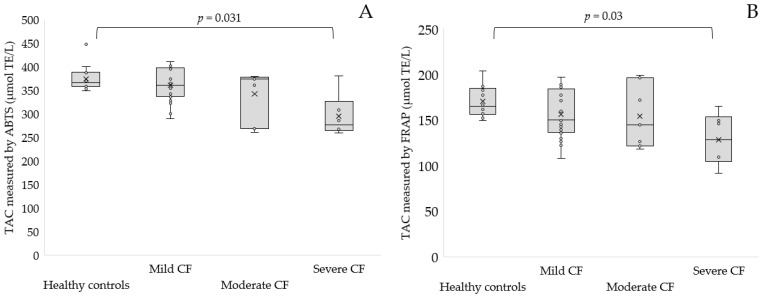
TAC level measured by ABTS^•^ (**A**) and TAC measured by FRAP (**B**) in the serum of patients with mild, moderate and severe CF as compared to the control group.

Furthermore, we found significantly lower TAC measured by ABTS^•^ and FRAP in severe CF patients with respect to healthy controls (294.96 ± 45.55 µmol TE/L for ABTS^•^, *p* = 0.031 and 128.68 ± 28.81 µmol TE/L for FRAP, *p* = 0.03, Figure 5A,B).

**Figure 6:**
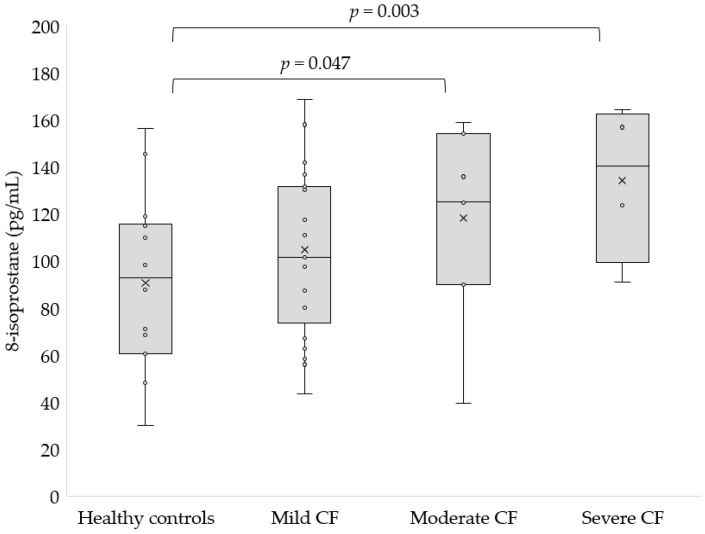
Concentration of 8-isoprostane in the serum of patients with mild, moderate and severe CF as compared to the control group.

Finally, we noted a significantly higher concentration of 8-isoprostane in severe (126.08 ± 29.58 pg/mL, *p* = 0.003) and moderate CF (118.27 ± 41.51 pg/mL, *p* = 0.047, Figure 6) compared to healthy controls.

Analysis of bacterial infection and oxidative stress markers revealed that there was no difference in the concentration of AOPP, Amadori products, TAC, lipid peroxidation markers and albumin cobalt binding between the study groups (Table 4).

A significantly higher 3-nitrotyrosine concentration was observed in CF patients infected with *P. aeruginosa* (*p* = 0.002) compared to healthy controls. Furthermore, we found a significantly decreased concentration of thiol groups in CF participants infected with *S. aureus* (*p* = 0.003). Additionally, we tried to assess the correlation between the estimated markers of oxidative stress and age, BMI, FEV_1_, white blood cells and CRP among the studied CF participants (Table 5).

We found moderate or weak significant positive correlations of AOPP (R = 0.47, *p* = 0.003), 3-nitrotyrosine (R = 0.373, *p* = 0.025) and MDA (R = 0.368, *p* = 0.025) with age. Furthermore, the concentration of thiol groups and the TAC measured by ABTS^•^ were inversely correlated with age. Additionally, BMI positively correlated with 3-nitrotyrosine (R = 0.333, *p* = 0.46). A moderately significant negative correlation (R = −0.493, *p* = 0.002) was observed between AOPP concentration and FEV_1_. Furthermore, 3-nitrotyrosine and 8-isoprostane were inversely correlated with FEV_1_. Additionally, moderately significant positive correlations of thiol group concentration and TAC measured by ABTS^•^ with FEV_1_ were observed. Surprisingly, there was no correlation between the markers of oxidative stress and white blood cells, as well as CRP concentration.

## 4. Discussion

Oxidative stress in CF patients is caused by both pulmonary and non-pulmonary symptoms of the disease [26]. Chronic respiratory tract infections lead to an excessive immune response mainly through a massive neutrophil invasion of the airways, the production of large amounts of ROS and the release of granules [27]. This is due to the activity of neutrophils, which trigger an oxidative burst to remove pathogens from the airways [28]. It is worth noticing that previous studies show abnormal or normal intrinsic reactive oxygen species generation in neutrophils of CF patients [29,30]. In addition, it has been shown that the modulation of biological processes related to survival functions in CF cell lines is mediated by oxidative stress [31]. The concentration of AOPP was non-significantly elevated in CF patients compared to controls, which is in the line with our previous study on pediatric patients [32]. Nevertheless, another marker of protein oxidation—carbonyl protein—was significantly (*p* < 0.05) increased in children with CF, which was previously reported by us [33]. Low levels of glutathione (GSH) were reported in CF patients, which may be the cause of decreased concentrations of thiol groups [34,35]. Furthermore, a defective CFTR channel has been shown to cause a decrease in both extracellular and intracellular GSH levels, possibly by reducing the amount of extracellular cystine required for the synthesis of GSH [36]. Similarly to our results, an increased level of 3-nitrotyrosine was found in exhaled breath condensate and sputum of CF patients, reflecting the formation of reactive nitrogen intermediates [37,38]. Nonetheless, no difference in the 3-nitrotyrosine concentrations between the young CF patients and healthy subjects was also reported by Celio et al., suggesting that oxidative stress increases with age [39]. The formation of oxidizing and possibly nitrating species derived from myeloperoxidase in the airways of patients with CF, collectively, may contribute to bronchial injuries and respiratory failure in CF [38]. To support the concept that neutrophil oxidants are involved in deterioration of lung function, myeloperoxidase activities are elevated in the sputum and bronchoalveolar liquid in patients with CF [40]. Furthermore, a study on CFBE41o-cells expressing F508del CFTR revealed increased activity of NADPH oxidase and expression level, mainly responsible for the elevated ROS production [36]. TAC was lowered in CF patients; however, the difference was not statistically significant (2.02 ± 1.08 mmol TE/L vs. 2.35 ± 0.93 mmol TE/L in controls) [41]. Similar values of TAC were observed in a study by Olveira et al. among CF patients > 16 years of age [42]. Significantly decreased TAC was observed in CF pediatric patients with respect to controls in a study by Sadowska-Woda et al. [43]. Lands et al. reported that the TAC values were similar between controls and CF patients who were not hospitalized. In contrast, when the patient was hospitalized, the TAC was significantly lowered [44].

MDA, which is the product of lipid peroxidation, was higher in adults with CF compared to controls [41]. Similarly, elevated MDA level was observed in patients’ plasma, sputum and exhaled breath condensate [10,33,42]. An increased concentration of 8-isoprostane was also found in adult CF patients in a study by Olveira et al. They found that the serum level of 8-isoprostane was 111.68 ± 34.55 pg/mL compared to 88.08 ± 29.17 pg/mL (*p* < 0.05) estimated in controls [41]. Another study reported similar values of 8-isoprostane concentration reported in CF and control participants (111.6 ± 99.8 vs. 88.7 ± 29 pg/mL, *p* < 0.001) [42]. Increased level of 8-isprostane was also reported in exhaled breath condensate of children with CF [33].

To our knowledge, this is the first report showing 4-HNE levels in serum of CF patients, which was very similar to healthy participants, which suggests that not all polyunsaturated fatty acids are oxidized to the same extent in CF. However, an elevated concentration of 4-HNE was noted in chronic obstructive pulmonary disease and pulmonary fibrosis, leading to the imbalance of the expression of both proinflammatory mediators and protective antioxidant genes [45,46]. Moreover, to our knowledge, this is also the first study to present albumin cobalt binding estimation. Albumin cobalt binding test revealed a decreased ability of serum albumin to bind cobalt ions in CF patients, which may be associated with a decrease in albumin concentration in CF patients [47]. Similarly, the level of vitamin D binding protein was also decreased in both clinically stable CF and subjects with CF exacerbation [47].

Increased oxidative stress is associated with the severity of disease. In our study, as the severity of disease increased, we observed a drop in the thiol groups and TAC levels, as well as a growth in the concentration of 3-nitrotyrosine and 8-isoprostane. Likewise, a significantly lower concentration of thiol groups was noted in mild-to-moderate CF in respect to participants with severe pulmonary dysfunction (582.4 vs. 514.7 μmol/L, *p* = 0.013) [48]. Contrary to our results, patients with mild-to-moderate CF had a lower concentration of plasma MDA compared to MDA level in severe CF (153.4 ± 21.2 vs. 201.2 ± 23.1 nmol/L, *p* < 0.05) [10]. Similarly, an increased concentration of MDA was noted in severe CF participants compared to mild-to-moderate pulmonary dysfunction (9.28 vs. 8.14 μmol/L, *p* = 0.074) [48]. The reason for the lack of statistical significance among other markers may be the small number of patients in the group with moderate and severe disease.

It might seem that bacterial infection leads to oxidative stress, which propagates the inflammatory response, especially in the case of *P. aeruginosa* infection [49]. Moreover, co-infection by the two species is a frequent situation that may promote their interaction [1]. However, we did not find any differences in AOPP between the studied groups of CF patients divided according to bacterial infection, which is in agreement with our previous report [32]. Nevertheless, elevated carbonyl group content was previously reported by us in *P. aeruginosa* and *S. aureus* infected CF patients (*p* < 0.05) [32]. The oxidation products of GSH were elevated in bronchoalveolar lavage of CF children with pulmonary infections with *P. aeruginosa* compared to those without (*p* < 0.05) [50]. In our study, the level of thiol groups was significantly reduced in patients with CF infected with *S. aureus* compared to healthy subjects. Moreover, the activity of myeloperoxidase and the levels of halogenated tyrosines were higher in children with *P. aeruginosa* infection [51]. Similar to our results, there was no difference in MDA levels between patients infected with *P. aeruginosa* and other bacteria [10]. It is worth highlighting that a recent study reported increased susceptibility of *P. aeruginosa* to antibiotics and reduced pathogenicity in mice with elevated oxidative stress [52].

We found several correlations between the markers of oxidative stress and general characteristics of patients in this study. Concentration of AOPP, thiol groups, 3-nitrotyrosine and TAC estimated by the method with ABTS^•^ and MDA were correlated with age. BMI was associated only with 3-nitrotyrosine, while FEV_1_ was correlated with AOPP, thiol groups, 3-nitrotyrosine and TAC estimated by the method with ABTS^•^ and 8-isoprostane. None of the markers correlated with white blood cells and CRP. Nevertheless, protein carbonyls were not associated with age in the bronchoalveolar lavage fluid of CF children [40]. The extent of protein oxidation was inversely correlated with lung function, which is in line with our results and directly related to neutrophil granulocyte count [53]. Contrary to our studies, a study by Balint et al. reported strong positive correlation between 3-nitrotyrosine in exhaled breath condensate and FEV_1_ (R = 0.70, *p* < 0.0001) [37]. Moreover, unlike our results, TAC was positively correlated with anthropometric values (height, weight, BMI) but not with age in a study by Lands et al. However, similarly to our results, TAC and FEV_1_ were correlated (R = 0.43, *p* = 0.02) [44]. Subsequently, no significant correlations were observed between the plasma MDA values and lung function or differential cell counts, which is in line with our results [10]. Nevertheless, a significant negative correlation (R = −0.43, *p* < 0.05) between MDA and FEV_1_ was described in a study by Brown et al. [48]. A strong inverse correlation (R = −0.76, *p* < 0.05) between 4-HNE and FEV_1_ was reported in lungs of patients with chronic obstructive pulmonary disease [45]. Afterward, a study by Kelk et al. indicated that, in patients with CF, there were no increase in ROS production on hospital admission for acute pulmonary exacerbation. Moreover, there were no associations between ROS production and high-sensitivity CRP in either children or adults with CF [54]. Simultaneously, it should be noted that lower levels of vitamins, other antioxidants and antioxidant enzymes were observed in CF patients not only in the serum or plasma but also in the bronchoalveolar lavage, which indicates a reduced ability to neutralize ROS [40,41,55,56]. Antioxidant-enriched multivitamin treatment was associated with a lower risk of first pulmonary exacerbation, which also resulted in increased systemic antioxidant concentrations and reductions in systemic inflammation [57]. Nevertheless, despite a significantly higher intake of fat-soluble antioxidant vitamins, it was not possible to counteract ROS production, as the serum levels of these antioxidants were not sufficiently increased, which was also confirmed in our study [56].

Although our study presents many interesting findings, including the increase in oxidative stress with the severity of the disease and estimation of 4-HNE, as well as the ability of albumin to cobalt bind in CF, several limitations should be mentioned. First, it was a single-center study involving 42 patients. Second, the number of patients in the moderate and severe CF group was small. Additionally, we did not analyze the patients’ diets, including the intake of vitamins and other antioxidants. Moreover, our study results cannot be generalized to the entire CF population due to the exclusion criteria that were used in the study.

## 5. Conclusions

In summary, elevated levels of oxidative stress, including products of protein and lipid oxidation, as well as decreased total antioxidant capacity, were found in the serum of people with CF. Moreover, the level of oxidative stress increased with age and severity of the disease. The presence of bacterial infection with *P. aeruginosa* or *S. aureus* only had an effect on thiol groups and 3-nitrotyrosine levels, while co-infection by two species did not affect the level of oxidative stress, which may suggest that oxidative stress is not affected by bacterial infection in CF. Finally, it is worth adding that despite the intake of vitamins and nutritional drinks, oxidative stress occurs in patients with CF.

## Figures and Tables

**Table 1:** Participant demographics at enrollment ^a^.

		CF	Healthy Controls	*p*
Sex (F/M)		20/22	10/6	
Age (years)	mean ± SD	19.24 ± 7.92	19.25 ± 7.3	0.996
	range	10–39	10–38	
Genotype				
Homozygous ΔF508, *n* (%)		32 (76.2)	–	–
Heterozygous ΔF508, *n* (%)		10 (23.8)	–	–
Anthropometric measurements				
Height (cm)	mean ± SD	156.92 ± 17.92	160.56 ± 15.05	0.497
	range	124–188.5	130–180	
Weight (kg)	mean ± SD	49.27 ± 13.12	58.75 ± 13.89	0.023
	range	22.1–76	34–82	
BMI (kg/m^2^)	mean ± SD	19.73 ± 2.79	22.47 ± 2.52	0.001
	range	14.37–25.92	18.67–25.59	
Clinical laboratory markers				
WBC (10^3^/µL)	mean ± SD	9.93 ± 3.48	7.46 ± 2.32	0.018
	range	5.05–19.26	4.28–10.47	
NEU (%)	mean ± SD	61.03 ± 14.98	59.12 ± 6.08	0.58
	range	25.1–82.3	50.6–68.6	
LYMP (%)	mean ± SD	28.55 ± 13.67	31.42 ± 3.77	0.179
	range	8.7–66.9	25.4–38.8	
MONO (%)	mean ± SD	6.73 ± 1.69	6.81 ± 1.54	0.724
	range	3.3–12.4	4.3–9.7	
EOS (%)	mean ± SD	1.82 ± 1.83	1.04 ± 1.04	0.153
	range	0–8.4	0.2–4.2	
BASO (%)	mean ± SD	0.536 ± 0.25	0.344 ± 0.121	0.002
	range	0.2–1.3	0.2–0.5	
Na^+^ (mmol/L)	mean ± SD	138.1 ± 2.3	138.4 ± 1.5	0.518
	range	135–143	136–141	
K^+^ (mmol/L)	mean ± SD	4.22 ± 0.37	4.12 ± 0.25	0.485
	range	3.6–5.3	3.7–4.6	
Cl^−^ (mmol/L)	mean ± SD	103.6 ± 2.5	103.2 ± 2.1	0.412
	range	96–108	100–106	
CRP (mg/L)	mean ± SD	5.3 ± 4.6	1.92 ± 1.22	0.0008
	range	0.5–22	0.3–4.2	
Pulmonary function				
FVC (% predicted)	mean ± SD	92.1 ± 22.6	103.4 ± 5.8	0.02
	range	52–143	98–118	
FEV_1_ (% predicted)	mean ± SD	86.83 ± 25.1	102.4 ± 8.2	0.005
	range	35–142	97–127	
FEV_1_/FVC (% predicted)	mean ± SD	88.4 ± 17.9	100.7 ± 5.8	0.0002
	range	35–120	94–120	
Severity of disease				
Mild (FEV_1_ > 75%), *n* (%)		27 (64.3)	–	–
Moderate (FEV_1_ > 45%, <75%), *n* (%)		9 (21.4)	–	–
Severe (FEV_1_ < 45%), *n* (%)		6 (14.3)	–	–
Bacterial infection				
*P. aeruginosa*, *n* (%)		10 (23.8)	–	–
*S. aureus*, *n* (%)		13 (31)	–	–
Co-infected with *P. aeruginosa* and *S. aureus*, *n* (%)		9 (21.4)	–	–
Uninfected, *n* (%)		10 (23.8)	16 (100)	–

^a^ BMI—body mass index, WBC—white blood cells, NEU—neutrophils, LYMP—lymphocytes, EOS—eosinophils, BASO—basophils, CRP—C-reactive protein, FVC—forced vital capacity, FEV_1_—forced expiratory volume in 1 s; differences between means were analyzed using Mann–Whitney U-test using Statistica software (version 13.1, StatSoft Inc. 2016, Tulsa, OK, USA).

**Table 2:** Markers of oxidative stress in controls and participants with CF ^a^.

		Healthy Controls	CF	*p*
AOPP (nmol/mg protein)	mean ± SD	185.48 ± 59.42	228.06 ± 124.67	0.479
	range	122.89–294.26	123.09–552.26	
Thiol groups (μmol/L)	mean ± SD	591.65 ± 67.8	491.57 ± 86.34	0.0003
	range	478.93–738.11	357.92–749.77	
Amadori product (nmol/mg protein)	mean ± SD	1629.99 ± 269.36	1712.94 ± 346.35	0.741
	range	1023.75–1925.34	1185.15–2490.32	
ABTS (μmol TE/L)	mean ± SD	374.74 ± 25.89	347.27 ± 46.11	0.16
	range	349.09–447.52	259.74–410.58	
FRAP (μmol TE/L)	mean ± SD	170.68 ± 16.72	151.6 ± 28.92	0.023
	range	149.58–204.15	91.68–199.59	
4-HNE (pg/mL)	mean ± SD	346.52 ± 115.93	372.18 ± 143.13	0.501
	range	180.74–642.64	207.87–890.8	
Albumin cobalt binding (ABSU)	mean ± SD	0.28 ± 0.04	0.33 ± 0.07	0.038
	range	0.22–0.34	0.24–0.59	

^a^ AOPP—advanced oxidation protein products, ABTS—total antioxidant capacity measured by ABTS^•^, FRAP—total antioxidant capacity measured by FRAP, 4-HNE—4-hydroxy-2-nonenal; differences between means were analyzed using Mann–Whitney U-test using Statistica software (version 13.1, StatSoft Inc. 2016, Tulsa, OK, USA).

**Table 3:** Markers of oxidative stress in participants with CF depending on severity of illness ^a^.

		Mild CF (*n* = 27)	Moderate CF (*n* = 9)	Severe CF (*n* = 6)
AOPP (nmol/mg protein)	mean ± SD	191.74 ± 77.96	269.77 ± 173.31	324.65 ± 167.14
	range	132.09–455.93	123.09–552.26	144.48–506.45
Amadori product (nmol/mg protein)	mean ± SD	1683.4 ± 289.6	1739.45 ± 445.57	1817.57 ± 499.42
	range	1200.65–2490.32	1185.15–2388.5	1270.49–2346.46
MDA (μmol/L)	mean ± SD	3.33 ± 0.43	3.5 ± 0.43	4 ± 0.55
	range	2.48–4.25	2.99–4.12	3.16–4.48
4-HNE (pg/mL)	mean ± SD	319.93 ± 64.19	440.53 ± 199.71	510.17 ± 202.27
	range	207.87–441.54	251.46–734.05	314.43–890.8
Albumin cobalt binding (ABSU)	mean ± SD	0.34 ± 0.08	0.33 ± 0.07	0.32 ± 0.05
	range	0.24–0.59	0.27–0.45	0.26–0.4

^a^ AOPP—advanced oxidation protein products, MDA—malondialdehyde, 4-HNE—4-hydroxy-2-nonenal; differences between means were analyzed using Kruskal–Wallis test using Statistica software (version 13.1, StatSoft Inc. 2016, Tulsa, OK, USA).

**Table 4:** Markers of oxidative stress in participants with CF depending on bacterial infection ^a^.

		Healthy Controls	CF Infected with *P. aeruginosa* (*n* = 10)	CF Infected with *S. aureus* (*n* = 13)	CF Co-Infected with *P. aeruginosa* and *S. aureus* (*n* = 9)	CF Uninfected (*n* = 10)
AOPP (nmol/mg protein)	mean ± SD	185.48 ± 59.42	179.35 ± 53.21	255.34 ± 152.58	208.4 ± 120.11	255.68 ± 138.91
	range	122.89–294.26	132.09–289.18	123.09–552.26	139.93–473.25	130.48–506.45
Thiol groups (mmol/L)	mean ± SD	591.65 ± 67.8	497.09 ± 99.34	463.91 ± 68.29 *	494.54 ± 65.08	517.56 ± 109.03
	range	478.93–738.11	357.92–634.1	373.92–594.91	375.66–579.71	393.96–749.77
Amadori product (nmol/mg protein)	mean ± SD	1629.99 ± 269.36	1635.47 ± 345.13	1875.54 ± 398.33	1654.22 ± 301.84	1637.33 ± 294.04
	range	1023.75–1925.34	1185.15–2169.71	1200.65–2490.32	1270.49–2189.56	1242.65–1960.67
3-nitrotyrosine (nmol/mg protein)	mean ± SD	0.11 ± 0.01	0.14 ± 0.03 *	0.12 ± 0.01	0.13 ± 0.03	0.12 ± 0.01
	range	0.08–0.13	0.11–0.2	0.11–0.15	0.1–0.19	0.11–0.14
ABTS (μmol TE/L)	mean ± SD	374.74 ± 25.89	349.46 ± 34.25	332.48 ± 50.09	349.21 ± 51.91	363.28 ± 47.96
	range	349.09–447.52	290.02–402.37	260.92–399.84	269.23–405.45	259.74–410.58
FRAP (μmol TE/L)	mean ± SD	170.68 ± 16.72	349.46 ± 34.25	332.48 ± 50.09	349.21 ± 51.91	363.28 ± 47.96
	range	149.58–204.15	290.02–402.37	260.92–399.84	269.23–405.45	259.74–410.58
MDA (μmol/L)	mean ± SD	3.17 ± 0.28	3.57 ± 0.48	3.36 ± 0.59	3.74 ± 0.51	3.3 ± 0.32
	range	2.77–3.72	3.16–4.48	2.48–4.42	2.99–4.21	2.97–3.85
4-HNE (pg/mL)	mean ± SD	346.52 ± 115.93	426.14 ± 178.36	356.64 ± 100.66	333.03 ± 73.83	369.69 ± 188.39
	range	180.74–642.64	246.85–734.05	207.87–528.93	251.46–441.54	208.4–890.8
8-isoprostane (pg/mL)	mean ± SD	85.18 ± 37.33	108.53 ± 37.41	116.64 ± 33.24	112.07 ± 38.97	104.6 ± 39.38
	range	30.01–156.18	43.62–154.12	58.3–164.24	39.45–158.82	56.06–168.78
Albumin cobalt binding (ABSU)	mean ± SD	0.28 ± 0.04	0.31 ± 0.05	0.35 ± 0.09	0.35 ± 0.07	0.32 ± 0.06
	range	0.22–0.34	0.27–0.4	0.24–0.59	0.28–0.45	0.27–0.43

^a^ AOPP—advanced oxidation protein products, ABTS—total antioxidant capacity measured by ABTS^•^, FRAP—total antioxidant capacity measured by FRAP, MDA—malondialdehyde, 4-HNE—4-hydroxy-2-nonenal; differences between means were analyzed using Kruskal–Wallis test using Statistica software (version 13.1, StatSoft Inc. 2016, Tulsa, OK, USA), * *p* < 0.01 vs. control.

**Table 5:** Spearman’s rank correlation coefficients and *p* values between oxidative stress parameters and general characteristics of patients ^a^.

	Age		BMI		WBC		FEV_1_		CRP	
	*R*	*p*	*R*	*p*	*R*	*p*	*R*	*p*	*R*	*p*
AOPP	0.47	0.003	0.24	0.153	0.155	0.359	−0.493	0.002	0.108	0.525
Thiol groups	−0.449	0.006	0.172	0.315	−0.215	0.208	0.516	0.001	−0.01	0.954
Amadori products	0.149	0.387	0.134	0.437	−0.183	0.285	−0.054	0.752	−0.199	0.245
3-nitrotyrosine	0.373	0.025	0.335	0.046	0.050	0.771	−0.347	0.038	−0.246	0.148
TAC (ABTS)	−0.373	0.023	−0.104	0.54	0.220	0.190	0.48	0.003	0.072	0.673
TAC (FRAP)	−0.138	0.416	−0.206	0.221	−0.007	0.967	0.22	0.191	−0.1	0.555
MDA	0.368	0.025	0.147	0.385	−0.124	0.463	−0.252	0.133	−0.048	0.776
4-HNE	0.217	0.19	0.112	0.503	−0.077	0.644	−0.251	0.129	−0.113	0.499
8-Isoprostane	0.279	0.09	0.221	0.183	0.166	0.321	−0.395	0.014	−0.017	0.92
Albumin cobalt binding	−0.07	0.676	0.251	0.128	−0.040	0.814	0.091	0.585	−0.146	0.382

^a^ BMI—body mass index, WBC—white blood cells, FEV_1_—forced expiratory volume in 1 s, CRP—C-reactive protein, AOPP—advanced oxidation protein products, ABTS—total antioxidant capacity measured by ABTS^•^, FRAP—total antioxidant capacity measured by FRAP, MDA—malondialdehyde, 4-HNE—4-hydroxy-2-nonenal; Spearman’s rank correlation coefficients and *p* values were estimated using Statistica software (version 13.1, StatSoft Inc. 2016, Tulsa, OK, USA).

## Data Availability

Data supporting the results of this study shall, upon appropriate request, be available from the corresponding author.
